# Research hotspots and frontier analysis on Mendelian randomization in osteoporosis-related fields: A review based on bibliometric and visual analysis

**DOI:** 10.1097/MD.0000000000041961

**Published:** 2025-04-11

**Authors:** Qingqing Zeng, Sijie Gui, Zhuolan Li, Fei Wu, Dan Peng, Guqing Zeng

**Affiliations:** a School of Nursing, University of South China, Hengyang, China; b Jiangbei Campus of The First Affiliated Hospital of Army Medical University (The 958th Hospital of Chinese People’s Liberation Army), Chongqing, China; c Department of Orthopedics, The Second Xiangya Hospital of Central South University, Changsha, China.

**Keywords:** bibliometric, hotspots, mendelian randomization, osteoporosis, visual analysis

## Abstract

This research seeks to thoroughly examine the present state and research hotspots in Mendelian randomization (MR) in osteoporosis (OP)-related fields, providing a reference for future research directions in this area. Following an exhaustive search of the Web of Science Core Collection database, our analysis utilized citation statistics for osteoporosis studies conducted from January 1, 2007, through March 15, 2024. Bibliometric and visual analyses were conducted using two online analysis systems, CiteSpace and VOSviewer. The analysis included a variety of dimensions, including the distribution of citations, authorship, published journals, year of publication, countries, co-occurrence keywords and keyword clustering. From 2007 to 2024, the number of publications in MR in OP-related fields showed an overall increased. High-impact publications and leading contributors were primarily from China, followed by the USA and England. The journal with the largest number of publications in MR in OP-related fields is the Journal of Bone and Mineral Research. Risk factor research, genome-wide associations, meta-analysis, fractures, and gene loci were the main research hotspots. Interdisciplinary integration, MR research combined with data from clinical trials and observational studies, represents the future development trend. Research on MR in OP-related fields has mainly been conducted in China, the USA, and England. The research hotspots were aimed at exploring the causative between risk factors and OP. Future studies are likely to center on multidisciplinary integration.

## 1. Introduction

Osteoporosis (OP) is a condition affecting bones, marked by low bone mineral density and deteriorated bone microstructure, which poses a major worldwide public health challenge and leads to substantial economic costs for societies globally.^[1–3]^ OP is a complex multifactorial disease, influenced by various factors, and results from a complicated interaction among biological, behavioral, and environmental elements.^[4]^ These biological aspects encompass genetic susceptibility, gender, age, and status following menopause. Behavioral and environmental factors encompass a low-calcium diet, reduced physical activity, limited outdoor engagement, smoking, and excessive alcohol intake.^[5]^ Therefore, it is essential to thoroughly explore its influencing factors.

Past research investigating osteoporosis has primarily focused on identifying risk factors and understanding their role in disease onset and progression.^[6,7]^ Numerous studies have used conventional epidemiological methods to explore factors such as calcium intake, physical activity, and smoking. However, these studies often fail to establish causal relationships due to confounding factors and biases.^[8,9]^ More recently, Mendelian randomization (MR) has emerged as a promising tool to overcome these limitations, as it uses genetic variants as instrumental variables to infer causal relationships between risk factors and outcomes.^[10,11]^ MR studies have provided new insights into the genetic basis of osteoporosis, identifying several genetic variants associated with bone mineral density and fracture risk. These findings have advanced our understanding of the disease’s etiology and may inform new prevention and treatment strategies.

While randomized controlled clinical trials (RCTs) are traditionally considered as the benchmark for investigating disease causality, the feasibility is often hindered by high costs, time constraints, and limited intervention options. MR has risen as a substitute methodology, using genetic variations as instruments to explore the causal connections between risk elements and outcomes. The field of genome-wide association studies has seen substantial evolution, culminating in the discovery of numerous genetic variants linked to phenotypes. As a result, MR methods have become increasingly popular for investigating etiological associations.

Bibliometric visualization analysis helps to identify the key hotspots and frontiers of disciplinary development by measuring the literature in a specific field.^[12]^ CiteSpace and VOSviewer software are frequently utilized for data visualization across various fields.^[13–15]^ This paper aims to fill this gap by conducting a bibliometric analysis of the MR literature in the context of osteoporosis. By employing CiteSpace and VOSviewer software, this study provides a comprehensive overview of the research hotspots and emerging trends in the field. The findings of this paper are particularly timely, as they will help guide future research directions and inform the design of studies that explore the causal relationships between genetic factors and osteoporosis. In addition, the insights gained may offer valuable information for the development of novel therapeutic approaches and prevention strategies, ultimately advancing the field of osteoporosis research.

## 2. Methods

### 2.1. Literature search

Our study involved a retrospective evaluation of publicly available data, which does not require approval from an institutional review board and does not involve experiments with human subjects or any animal samples. The literary collection of MR studies related to osteoporosis was searched and exported from the Web of Science Core Collection database. The earliest MR studies related to OP were published in 2007, marking the application of this approach in osteoporosis research. Consequently, we chose to initiate our search from January 1, 2007, to March 15, 2024.The search terms used were “Osteoporosis,” “Bone Mineral Density,” and “Mendelian Randomization.” The scope of the search was unrestricted, with the chosen literature being sourced from the Web of Science Core Collection database. Below is the methodology used to conduct the literature search:

1. “Osteoporosis” OR “Bone mineral density” OR “Senile Osteoporosis” OR “Age-Related Bone Loss” OR “Age-Related Osteoporosis” OR “Bone Loss” (all fields)
2. “Mendelian randomization study” OR “Mendelian Randomization Analysis” (all fields)
3. #1 AND #2

Imported search results into EndNote document management software for de-duplication and screening.

### 2.2. Data inclusion and exclusion

Inclusion criteria: Studies related to MR in osteoporosis-related fields, including research papers and reviews. Exclusion criteria: Letters, editorials, meeting abstracts, news reports, case reports, etc.

### 2.3. Data preprocessing and statistical analysis

Two authors independently conducted the searches, examined the publications, and retrieved the materials. In the event of a disagreement, a third author was consulted. Literature analysis deadline was March 15, 2024. We collected data on citations, authorship, published journals, year of publication, countries, and keywords. Furthermore, we independently reviewed literature, removed duplicate data, standardized the format, and assessed the keywords and author information. To ensure the quality and suitability of the data, 2 researchers conducted a comprehensive assessment, considering multiple dimensions such as research design, methodology, and data quality. This process ensures that our evaluation is as objective and accurate as possible. For manuscripts with authors from multiple countries, the credit was attributed to the country of the corresponding author listed in the manuscript. This approach ensures consistency in attributing research contributions and simplifies the scoring and analysis process.

The papers were carefully evaluated and categorized according to their title, author, country of origin, publishing journal, keywords, and abstract. Subsequently, the data were extracted and transferred into Excel as a.txt file. To investigate the relationships between the most frequently cited studies, CiteSpace (v.6.3.R1) was used, producing a cluster map for in-depth visual examination. CiteSpace software parameter setting: Time span close from January 2007 to March 2024, Years Per Slice selected “1.” Microsoft Excel 2021 (Microsoft Corporation, Redmond) was employed for statistical analyses and graph construction. We also utilized VOSviewer 1.6.20 for visual analysis, including the creation of a time distribution map, national cooperation distribution map, organization cooperation distribution map, keyword time zone map, literature citation map, word list, and more. VOSviewer software parameter setting: Visualization scale: 2.00, label size variation: 0.80, line size variation: 1.00, minimum strength:5.

## 3. Results

### 3.1. Analysis of the literature publications

**Figure 1.**
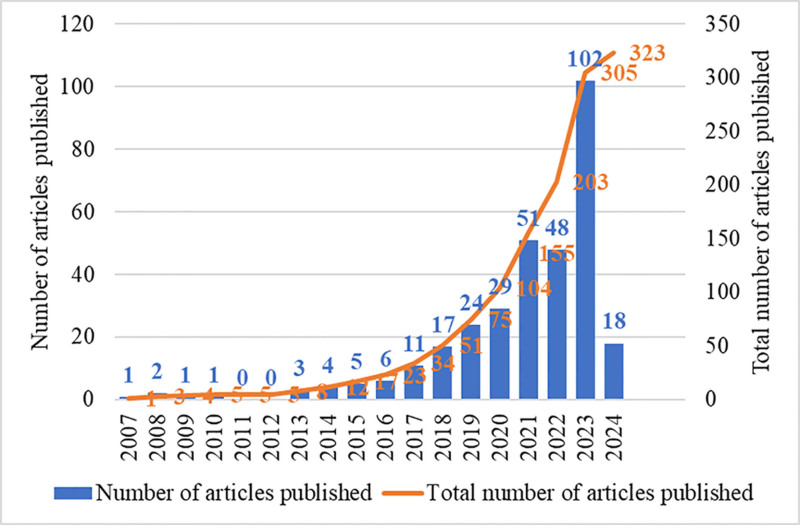
Trends in the volume of MR in OP-related fields articles from 2007 to 2024. MR = mendelian randomization, OP = osteoporosis.

A total of 357 articles were retrieved from the Web of Science Core Collection database. After screening, 323 articles were deemed to be relevant, consisting of 28 reviews and 295 original articles. The annual publication trends in this field reflect the developmental trajectory of MR in OP-related fields. The literature in MR in OP-related fields dates back to 2007, and subsequent research from 2007 to the present can be divided into 2 phases. During the first phase, spanning from 2007 to 2016, a total of 17 papers were published, accounting for 5.3% of the overall output. The relatively modest number of publications in MR in OP-related fields research indicated that relevant research in MR in OP-related fields was at a nascent stage. During the second phase, spanning from 2017 to 2024, 306 papers were published, accounting for a significant 94.7% of the total. Notably, the quantity of studies in MR in OP-related fields during this period exhibited a noteworthy increase annually. This suggested that the research field was rapidly expanding (Fig. 1).

### 3.2. Analysis of the publisher journals

This study included 323 articles from 116 journals. Table 1 showcased the top 10 journals with the highest number of publications, along with their most recent impact factors (IF). Leading the list in terms of publication frequency were the Frontiers in Endocrinology and Journal of Bone and Mineral Research, which contributed 27 and 23 articles, respectively. Additionally, the Frontiers in Endocrinology received the highest number of citations. According to the Journal Citation Reports (JCR), half of these journals are ranked in the Q1 quartile, with 39.8% categorized within the endocrinology metabolism field. Nature Genetics boasts the highest impact factor, standing at 30.8. Geographically, among the top 10 journals, 5 are located in the USA, 3 in England, and 2 in the Netherlands.

**Table 1:** Ranking of the top 10 major Journals of MR in OP-related fields articles from 2007 to 2024

Ranking	Sources	Articles	Percentage	Country	IF	JCR-c	Total link strength
1	Frontiers in endocrinology	27	8.36	Switzerland	5.2	Q1	68
2	Journal of bone and mineral research	23	7.12	USA	6.2	Q1	96
3	Journal of clinical endocrinology & metabolism	15	4.64	USA	5.8	Q1	32
4	Osteoporosis international	15	4.64	England	4.0	Q2	30
5	Calcified tissue international	14	4.33	USA	4.2	Q3	46
6	Frontiers in genetics	11	3.41	Switzerland	3.7	Q1	15
7	Bone	10	3.10	USA	4.1	Q2	46
8	Plos one	9	2.79	USA	3.7	Q2	18
9	Scientific reports	8	2.48	England	4.2	Q2	21
10	Frontiers in nutrition	8	2.48	Switzerland	5.0	Q1	9

IF = impact factor (2022–2023), JCR-c = Journal Citation Reports category (2023), MR = mendelian randomization, OP = osteoporosis.

### 3.3. Analysis of the most productive countries and institutions

**Figure 2.**
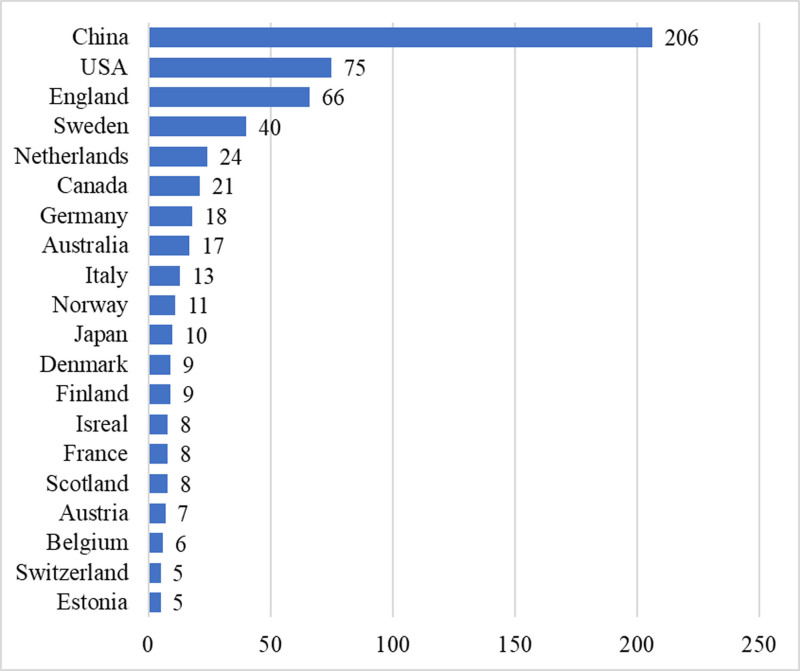
The number of articles on MR in OP-related fields publications by countries from 2007 to 2024. MR = mendelian randomization, OP = osteoporosis.

This study incorporated 323 articles originating from 42 distinct countries. The volume of articles from each country served as a measure of their respective contributions to the research area. Typically, a higher publication count reflected a more significant input. Figure 2 provided a detailed breakdown of the leading countries in articles related to MR in osteoporosis, with China at the forefront with 206 articles, followed by the USA with 75 and England with 66.

**Figure 3.**
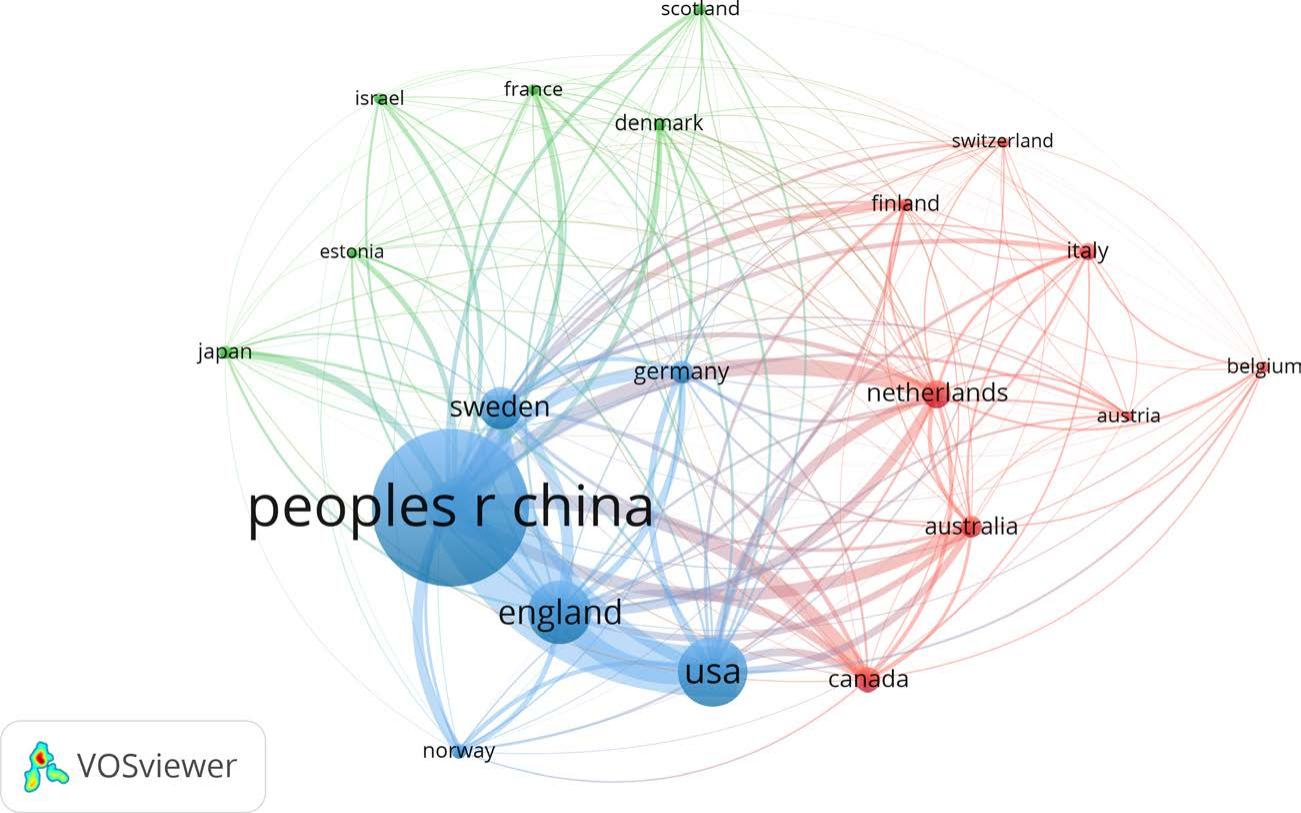
National collaborative network mapping of MR in OP-related fields publications from 2007 to 2024. MR = mendelian randomization, OP = osteoporosis.

Figure 3 showed the national collaborative network mapping visualized using VOSviewer, which displayed the distribution of countries contributions. Predominantly, research collaborations were observed among North America, Europe, Oceania, and East Asia. England was the country with the strongest links (178) to other countries in MR in OP-related fields of research, followed by the USA (173) and Sweden (125). The Figure 3 indicated that China and the USA were the foremost collaborators in research concerning MR in OP-related fields.

The 323 articles involved a total of 484 institutions. Table 2 displayed the top 10 institutions ranked by their publication count in descending order. Notably, the University of Bristol stood out for having the highest publication count (31) and the most extensive collaborations (43) with other institutions.

**Table 2:** Ranking of the top 10 institutions of MR in OP-related fields articles from 2007 to 2024

Ranking	Institutions	Articles	Percentage	Country	Total link strength
1	Univ Bristol	31	9.60	England	43
2	Tulane Univ	19	5.88	USA	29
3	Kings Coll London	18	5.57	England	32
4	Uppsala Univ	16	4.95	Sweden	24
5	Soochow Univ	16	4.95	China	12
6	Xi An Jiao Tong Univ	15	4.64	China	7
7	McGill Univ	14	4.33	Canada	38
8	Cent South Univ	13	3.10	China	17
9	Karolinska Inst	12	3.72	Sweden	24
10	Zhejiang Univ	12	3.72	China	14

MR = mendelian randomization, OP = osteoporosis.

### 3.4. Analysis of the most influential authors

A collective effort of 1771 authors led to the publication of 323 articles on MR in OP-related fields. Table 3 listed the top 10 authors based on the volume of articles they have published. The primary contributors to MR in OP-related fields were Dr George Davey Smith and Dr Hong-wen Deng, each with 14 articles, followed by Dr J. Brent Richards with 11 articles.

**Table 3:** Ranking of the top 10 major authors of MR in OP-related fields articles from 2007 to 2024

Ranking	Authors	Articles	Percentage	Total link strength	Location
1	Smith, George Davey	15	4.64	12	England
2	Deng, Hong-wen	14	4.33	31	USA
3	Larsson, Susanna C.	11	3.41	13	Sweden
4	Richards, J. Brent	10	3.10	21	USA
5	Ohlsson, Claes	10	3.10	14	Sweden
6	Zhang, Lei	8	2.48	14	China
7	Michaelsson, Karl	8	2.48	13	Sweden
8	Zheng, Jie	7	2.17	9	China
9	Wang, Wei	7	2.17	14	China
10	Tobias, Jonathan H.	7	2.17	8	England

IF = impact factor (2022–2023), JCR-c = Journal Citation Reports category (2023), MR = mendelian randomization, OP = osteoporosis.

### 3.5. Analysis of literature citations

We conducted a citation analysis of all referenced works and highlighted the main features of MR in OP-related fields research through the top 10 cited references from 2007 to 2024 (Table 4).^[16–25]^ Notably, 3 articles garnered upwards of 300 citations, and each of the top 10 received at least 123 citations. The most cited work, with 525 citations, was “Skeletal and Extraskeletal Actions of Vitamin D: Current Evidence and Outstanding Questions” by Bouillon, et al Among the 10 eminent articles in MR for osteoporosis, the one with the highest impact factor (82.9) came from Ruth, in 2020. More than half of the top 10 cited articles were published after 2017. Furthermore, England was the research location for 5 of these 10 notable articles.

**Table 4:** Ranking of the top 10 major co-citation of MR in OP-related fields articles from 2007 to 2024

Ranking	Authors (year)	Title	Country	IF	JCR-c	Citations	Total link strength
1	Bouillon, R. (2019)^[8]^	Skeletal and Extraskeletal Actions of Vitamin D: Current Evidence and Outstanding Questions	Belgium	20.3	Q1	525	9
2	Lips, P. (2019)^[9]^	Current vitamin D status in European and Middle East countries and strategies to prevent vitamin D deficiency: a position statement of the European Calcified Tissue Society	The Netherlands	5.8	Q1	368	1
3	Mokry, L. E. (2015)^[10]^	Vitamin D and Risk of Multiple Sclerosis: A Mendelian Randomization Study	Canada	15.8	Q1	309	14
4	Kemp, J. P. (2017)^[11]^	Identification of 153 new loci associated with heel bone mineral density and functional involvement of GPC6 in osteoporosis	Australia	30.8	Q1	290	50
5	Vimaleswaran, K. S. (2014)^[12]^	Association of vitamin D status with arterial blood pressure and hypertension risk: a mendelian randomization study	England	44.5	Q1	283	8
6	Ebrahim, S. (2008)^[14]^	Mendelian randomization: can genetic epidemiology help redress the failures of observational epidemiology?	England	5.3	Q1	243	7
7	Timpson, N. J. (2018)^[15]^	Genetic architecture: the shape of the genetic contribution to human traits and disease	England	42.7	Q1	233	1
8	O’Connor, L. J. (2018)^[16]^	Distinguishing genetic correlation from causation across 52 diseases and complex traits	USA	30.8	Q1	182	9
9	Lane, J. M. (2019)^[17]^	Biological and clinical insights from genetics of insomnia symptoms	USA	30.8	Q1	176	2
10	Ruth, K. S. (2020)^[13]^	Using human genetics to understand the disease impacts of testosterone in men and women	England	82.9	Q1	123	4

IF = impact factor (2022–2023), JCR-c = Journal Citation Reports category (2023), MR = mendelian randomization, OP = osteoporosis.

### 3.6. Analysis of the keywords

**Figure 4.**
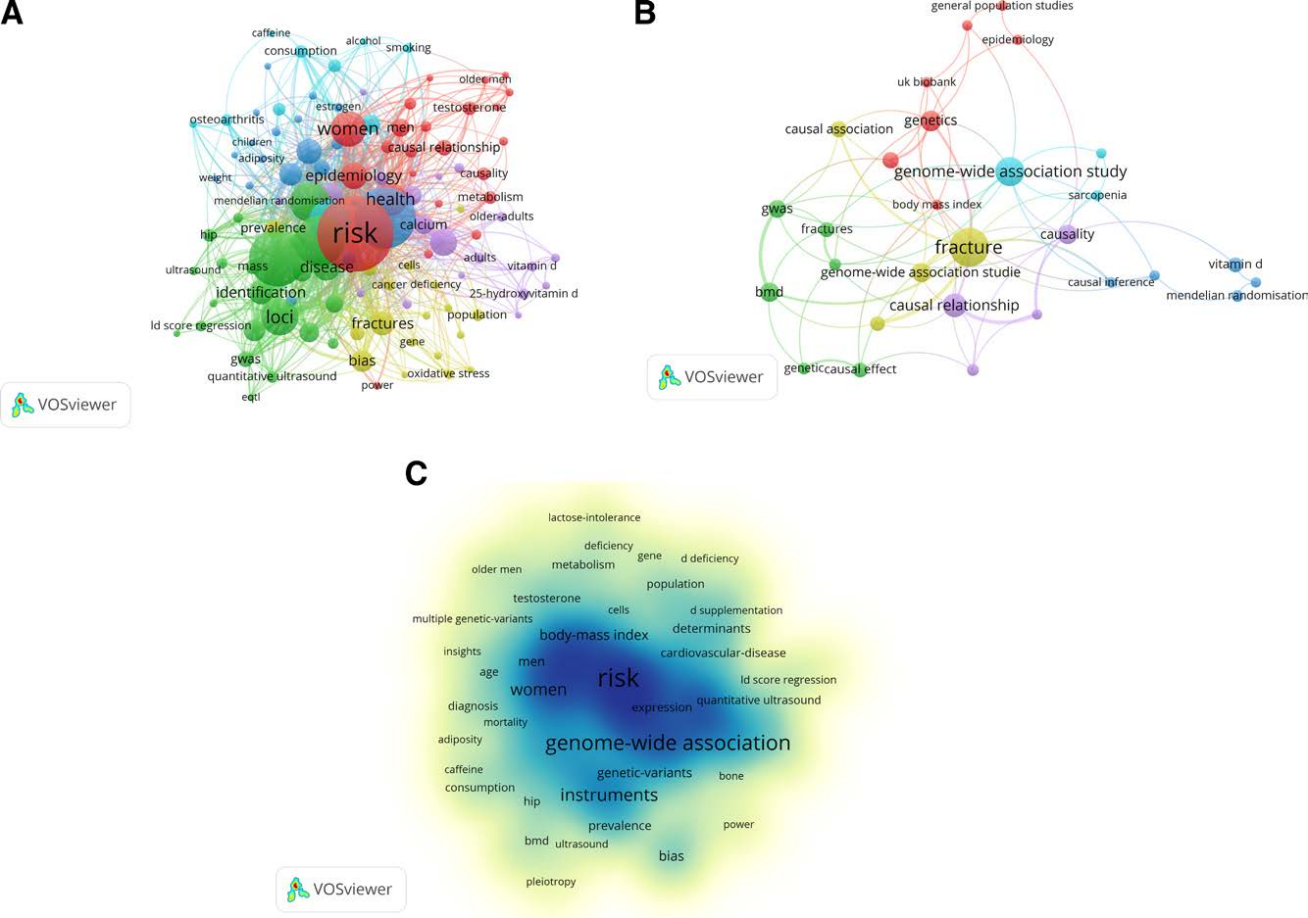
(A) keyword co-occurrence network map. (B) Authors keywords network map. (C) Density map of keyword-plus. MR = mendelian randomization, OP = osteoporosis.

**Figure 5.**
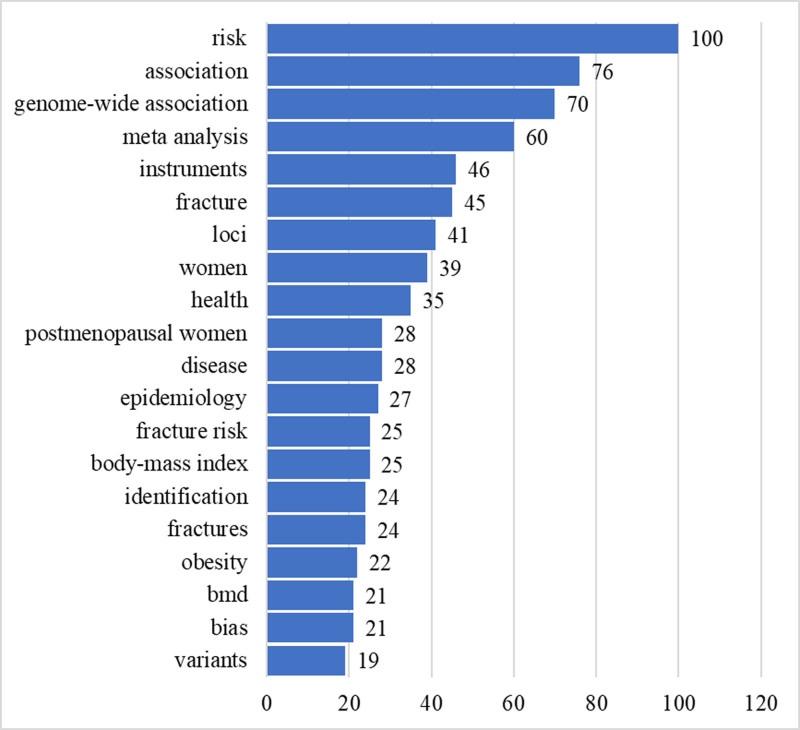
The keyword frequency of MR in OP-related fields articles from 2007 to 2024. MR = mendelian randomization, OP = osteoporosis.

Keyword analysis was a crucial bibliometric indicator. The analysis of Co-occurrence in publications reveals the interdependence of keywords.^[26]^ Following the standardization and consolidation of the initial keywords, a total of 1284 keywords were generated. Keywords related to the search strategy, such as “Mendelian randomization,” “osteoporosis,” and “bone mineral density” were removed. Keywords that appeared at least 5 times were compiled to construct a keyword co-occurrence network map, featuring 118 nodes (Fig. 4A). The primary terms identified by the author were centered around fractures and MR, including phrases like genome-wide association study, fracture, and genetic, as depicted in Figure 4B. Additionally, Keyword-plus terminology frequently encompassed terms associated with osteoporosis and aspects of the study’s aim, such as risk, fracture, and health, as shown in Figure 4C. The top 20 keywords occurrences are shown in Figure 5. The classification of words into high and low-frequency categories follows Zipf’s second law formula: $T=1/2\left(-1+\sqrt{1}+8\times I_{1}\right)$, where I_1_ signifies the count of keywords appearing only once in the dataset.^[27]^ Using this formula, keywords that occur 35 times or more are considered high-frequency, resulting in 8 high-frequency keywords. Prominent among the frequently used keywords were “risk” (n = 100), “association” (n = 76), “genome-wide association” (n = 70), “meta analysis” (n = 60), “instruments” (n = 46) “fracture” (n = 45), “loci” (n = 41), “women” (n = 39), “health” (n = 35).

**Figure 6.**
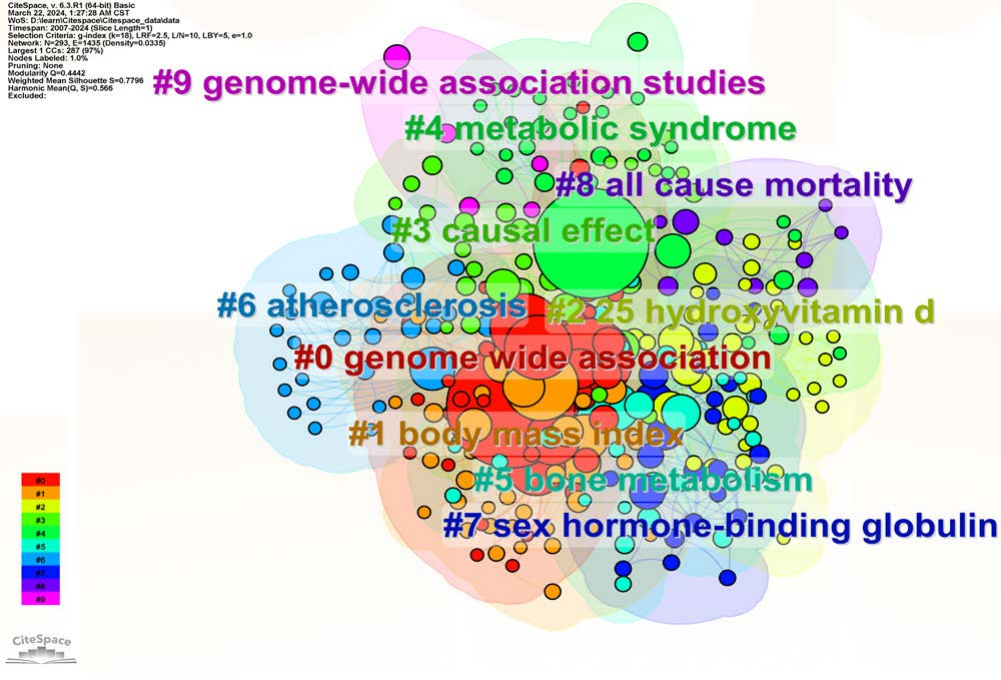
Keyword clustering network mapping of MR in OP-related fields articles from 2007 to 2024. MR = mendelian randomization, OP = osteoporosis.

**Figure 7.**
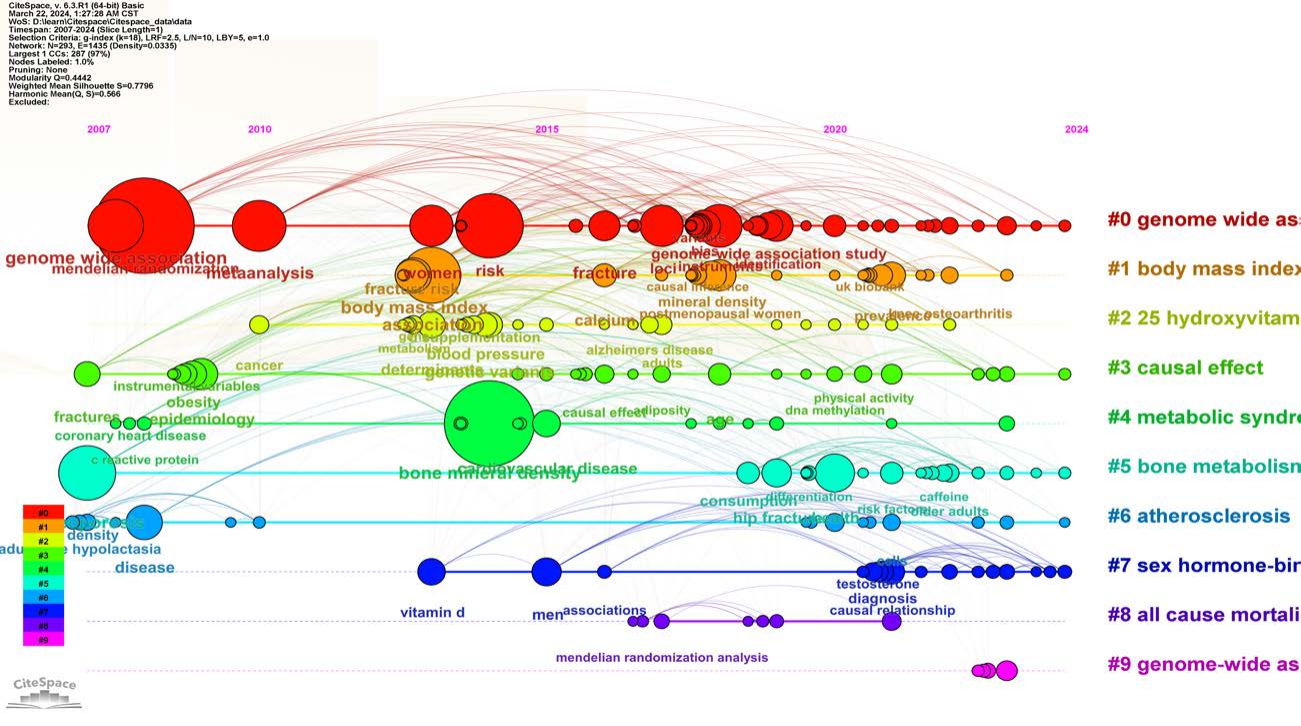
The keyword clustering timeline graph of MR in OP-related fields articles from 2007 to 2024. MR = mendelian randomization, OP = osteoporosis.

The map of keyword clustering was divided into 10 modules. The literature primarily concentrated in modules #0 to #9, identified as “#0 genome wide association,” “#1 body mass index,” “#2 25 hydroxyvitamin d,” “#3 causal effect,” “#4 metabolic syndrome,” “#5 bone metabolism,” “#6 atherosclerosis,” “#7 sex hormone-binding globulin,” “#8 all-cause mortality” and “#9 genome-wide association studies,” respectively (Fig. 6). Among the modules, the one most closely associated with exploring the factor influencing OP was “#3 causal effect.” The top 5 keywords scored by the Log-Likelihood Ratio (LLR) algorithm under module #3 were “causal effect” (13.57. 0.001), “epigenetic age” (10.07, 0.005), “inflammatory bowel disease” (6.42, 0.05), “two-sample mendelian randomization” (6.42, 0.05), “Crohn’s disease” (6.42. 0.05). Module #0 was closely related to MR research methods. The top 5 keywords scored by the LLR algorithm under module #0 were “genome wide association” (6.65, 0.01), “pleiotropy” (6.01, 0.05), “genome-wide association study” (5.92, 0.05), “meta-analysis” (5.88, 0.05), and “vitamin d” (5.7, 0.05). Module #2 was primarily associated with pathogenesis and drug targets. The top 5 keywords scored by the LLR algorithm under module #2 were “25 hydroxyvitamin d” (15.68, 1.0E-4), “25-hydroxyitamin d” (15.37, 1.0E-4), “blood pressure” (11.44. 0.001), “dementia” (10.23, 0.005), “d deficiency” (10.23, 0.005). As shown in the keyword clustering timeline graph (Fig. 7), module #7 sex hormone-binding globulin, has been a research hotspot in recent years.

## 4. Discussion

### 4.1. Current status of MR in OP-related fields research

OP has emerged as a major public health concern, and the rapid development of MR has introduced new perspectives for osteoporosis research. While bibliometric analysis is not the sole determinant of scientific quality, it serves as a valuable tool to quantify citation frequency and assess a paper’s impact within the field.^[28]^ In this research, we performed an in-depth examination of the evolution of MR in OP-related fields over the last seventeen years, utilizing tools such as CiteSpace and VOSviewer. Our dataset comprised 323 articles related to MR in OP-related fields. While MR in OP-related fields studies remained nascent from 2007 to 2016, there was a significant surge in research activity post-2016, indicating a growing significance in this research domain. In 2017, there was a surge in MR in OP-related fields studies. Specifically, in 2017, several researchers made significant contributions by elucidating the genetic predictors of inflammatory markers’ impact on osteoporosis etiology.^[29–31]^ This provides scholars with a deeper understanding of the mechanisms of related gene expression. The increase in studies after 2016 highlights a growing interest in exploring the cause-and-effect relationship between various factors and osteoporosis through genomic approaches.

Journals within the JCR database are organized and rated by quartile (Q) according to their IF within their specific fields. Q1 denotes the upper 25% of journals, showcasing the highest IF, and is followed in sequence by Q2, Q3, and Q4. Our analysis indicates that a significant portion of the leading 10 journals featuring research on MR in OP fall into the Q1 category (60%) and Q2 category (30%), demonstrating their esteemed status in the discipline. This notable presence in top-tier journals highlights the significant attention and recognition that MR in OP-related research has garnered. The core journals in this field were *Frontiers in endocrinology*, *Journal of Bone and Mineral Research*, and *Journal of Clinical Endocrinology & Metabolism*. These journals are high impact factor journals, suggesting that articles published in these journals are highly read and cited, which may elicit a greater influence on the field of osteoporosis. Future cutting-edge hotspots related to this field will likely be published in these journals.

The examination of the 1771 authors who contributed to the 323 articles on the research regarding MR and OP reveals a concentration of resources and collaborative efforts primarily in China, the USA, and England. This collaboration includes institutions such as the University of Bristol, Tulane University, and Uppsala University. England stood out for its extensive collaborative efforts with other nations, followed by the USA and Sweden. George Davey Smith had the highest number of publications of MR in OP-related fields, and his team was dedicated to exploring the causal relationship between body weight, lipids, and osteoporosis.^[32–34]^ This will not only help uncover new questions and research avenues but also allow for learning from their methodologies and empirical frameworks. Such insights and guidance will be valuable for advancing osteoporosis research. This ongoing attention and learning process will foster academic exchanges and collaboration, thus contributing to the overall development and progress of the field. China led in the volume of recent papers and had a notably close collaboration with the USA, which might be influenced by the increasing number of Chinese scholars pursuing education in the USA. This trend underscores the international scope of research activities in this area. To mitigate research limitations, recognizing the specific data sources, methodological boundaries, and the period of paper analysis is crucial. Providing this context enhances the study’s findings transparency and dependability.

### 4.2. Hotspots of MR in OP-related fields research

Combining high-frequency keywords and clustering results, relevant research hotspots and topics can be identified. Keyword co-occurrence analysis highlighted that “Risk” and “genome-wide association” were the most frequent, with a prominent hotspot focusing on OP risk factors explored through genomics. Keyword clustering identified 10 modules, with “genome wide association,” “25 hydroxyvitamin d,” and “causal effect” as prominent themes. Notably, the causal relationship between OP and sex hormone-binding globulin has emerged as a research hotspot. This indicates a shift towards understanding the broader implications of OP, extending beyond bone density to explore its connections with other health conditions. Especially, the first 3 modules were tightly interconnected, emphasizing the increasing interest in investigating the causal connections between OP and different diseases.

#### 4.2.1. Cause relationship between sex hormone-binding globulin and OP

Keyword clustering timeline graph display the frequency of keywords and their relationships in different time periods, providing an intuitive method for analyzing and understanding temporal correlation patterns in large amounts of text data. According to the analysis, the keyword clustering timeline graph, the research hotspots of MR in OP-related fields in recent years focused on sex hormone-binding globulin (SHBG). Four studies have examined the relationship between sex hormone-binding globulin and OP.^[35–38]^ SHBG, a sex hormone transport protein, can affect the level of sex hormone activity in the body by binding to sex hormones, which can impact bone density and health. These studies provide insights into the role of SHBG in the pathogenesis of osteoporosis, which will allow for more targeted solutions for individualized treatment strategies and sex hormone replacement therapy.^[35–38]^ Additionally, these studies will provide insights into the relationship between gender differences and the incidence of osteoporosis, leading to a more comprehensive understanding and solutions for the prevention and treatment of osteoporosis. Further studies are necessary to explore the role of sex differences in sex hormone regulation, SHBG levels, and bone metabolic processes. This will provide additional evidence for a deeper understanding of sex differences in osteoporosis.

#### 4.2.2. Causal relationship between 25 hydroxyvitamin D and OP

Keyword clustering analysis revealed that 25 hydroxyvitamin D was primarily associated with pathogenesis and drug targets. Several studies have explored the relationship between 25-hydroxyvitamin D and OP.^[39,40]^ This research area holds significance due to the essential role of vitamin D in preserving bone health and regulating calcium balance. These studies will enhance our understanding of the role of 25-hydroxyvitamin D in osteoporosis pathogenesis and the impact of vitamin D supplementation on bone health. These findings have significant implications for optimizing vitamin D use and developing more effective prevention and treatment strategies.

#### 4.2.3. Causal relationship between bone metabolism and OP

Keyword clustering analysis revealed that bone metabolism was strongly associated with other modules. Bone metabolism encompasses the processes involving osteoblast activity, calcium, and phosphorus metabolism within bone tissue, playing a pivotal role in maintaining bone density and overall bone health. Four studies related to bone metabolism and osteoporosis have been published in the last 2 years.^[38,41–43]^ The studies conducted in this area offer valuable insights into the pathogenesis of osteoporosis and the regulatory mechanisms of bone metabolism. While these findings provide a foundational understanding, they serve as a theoretical groundwork for the potential development of novel therapeutic and interventional approaches. By examining bone cell activity, bone matrix synthesis, and bone regeneration, we can advance our strategies for preventing and managing osteoporosis and related fractures^[44]^ Furthermore, the investigation of bone metabolism regulators introduces fresh perspectives and avenues for personalized treatment and intervention strategies.

These research foci are significant in enhancing our comprehension of the underlying mechanisms, prevention, and management of osteoporosis.

These studies contribute to a deeper understanding of the genetic contributions to osteoporosis, the influence of environmental factors on bone health, and the exploration of potential new treatment strategies. These findings may have a positive impact on future clinical practice, drug development and public health policy.

### 4.3. Research trends in MR in OP-related fields

Our focus is on recent research trends over the past 2 years. Specifically, we highlight 2 recently published studies that have explored the association between air pollutant exposure and osteoporosis.^[45,46]^ These studies suggest a potential link between air pollutants (e.g. PM2.5) and osteoporosis, contributing to our understanding of the possible impact of environmental factors on bone density and health. The findings underscore the importance of considering environmental factors in the prevention of osteoporosis and may inform the development of environmental protection policies and strategies to improve air quality. Future studies can further investigate the potential effects of various air pollutants on bone health, including PM2.5, PM10, NOx, and other pollutants. This could provide a more solid scientific foundation for preventive measures and treatments for osteoporosis, while promoting both public health and environmental protection. This emerging association is expected to become an important area of research in the field.

The results of our study indicated that MR was an effective approach for assessing the causality of osteoporosis. MR helped researchers determine the true drivers of osteoporosis and better-defined prevention and treatment strategies. It also distinguished between the contribution of genetic and environmental factors to the development of osteoporosis, deepening the understanding of the disease’s etiology. Moreover, by verifying causality, MR assisted in the development of individualized prevention and treatment protocols, ultimately improving patient outcomes and prognosis. In the field of osteoporosis research, MR studies have revealed significant insights, including the identification of specific genetic markers that could predict treatment efficacy, a crucial step towards personalized medicine in OP management. However, MR studies often focus on a single factor, leading to an incomplete understanding of the disease’s pathogenesis. To address these, we should combine MR findings with data from clinical trials and observational studies. It is crucial to integrate MR with genetic, bioinformatics, proteomics, and molecular biology in future research. This interdisciplinary approach can deepen our understanding of how genetic factors influence individual responses to OP treatments.

Adopting this holistic strategy provides a broader perspective on managing and treating osteoporosis. This approach enhances our understanding of the disease and facilitates the development of specialized prevention techniques and treatment options. A comprehensive approach can also emphasize the importance of lifestyle factors, such as calcium intake, weight-bearing physical activity, and sun exposure, particularly during childhood and young adulthood. These factors may help prevent bone loss before it occurs, reducing the need for treatment later in life. Ultimately, this strategy could lead to a future where osteoporosis management is highly personalized, significantly improving the quality of life for those affected by this condition. This marks a significant shift in osteoporosis research, moving us closer to the ultimate goal of precision medicine.

In summary, leveraging MR in conjunction with other research methodologies offers new opportunities for understanding and managing osteoporosis, particularly in the development of personalized treatment protocols.

## 5. Limitations

This study has several limitations. Initially, this study only analyzed papers from the Web of Science Core Collection database. Additionally, only English-language studies were selected, excluding potentially relevant research published in other languages. Moreover, because the database undergoes regular updates, the data for our bibliometric analysis might not perfectly reflect the current state of research, although the general patterns are expected to be consistent. The elevated rate of citations could be due to the phenomenon of self-citation, which involves authors citing their own work. Lastly, the tools and methods used for bibliometric visualization and analysis are still evolving, necessitating ongoing efforts to refine and standardize these approaches.

## 6. Conclusion

In this study, we provided a bibliometric review focusing on MR in osteoporosis-related fields. Through comprehensive analysis of relevant articles, we explored key research trends, including genetic factors, environmental influences, and the role of MR in understanding osteoporosis. We found that MR is an effective tool for assessing the causality of osteoporosis and offers valuable insights into personalized treatment strategies. This study also highlighted emerging trends, such as the potential impact of environmental factors like air pollution and the importance of early lifestyle interventions. Our findings suggest that a holistic, interdisciplinary approach that integrates MR with other methodologies, such as clinical trials and bioinformatics, is critical to advancing osteoporosis research and developing personalized treatment protocols. Interdisciplinary collaboration will be essential to unraveling the complex genetic underpinnings of osteoporosis and developing more effective prevention and treatment strategies.

## Acknowledgments

We would like to thank the School of Nursing, Hengyang Medical School, University of South China; University of South China-Hunan Lantern Medical Technology Co, Ltd School-Enterprise Cooperative Innovation and Entrepreneurship Education Base, Hengyang, Hunan Province, China.

## Author contributions

**Conceptualization:** Qingqing Zeng, Sijie Gui, Zhuolan Li.

**Data curation:** Qingqing Zeng, Zhuolan Li.

**Formal analysis:** Qingqing Zeng, Sijie Gui, Zhuolan Li.

**Methodology:** Qingqing Zeng, Sijie Gui, Zhuolan Li.

**Software:** Qingqing Zeng.

**Supervision:** Fei Wu.

**Validation:** Dan Peng, Guqing Zeng.

**Visualization:** Qingqing Zeng.

**Writing – original draft:** Qingqing Zeng.

**Writing – review & editing:** Qingqing Zeng, Guqing Zeng.
